# Investigating the role of the left inferior frontal gyrus in language evolution: insights from comparative neuroscience

**DOI:** 10.3389/fnhum.2025.1726577

**Published:** 2026-01-14

**Authors:** Jinyi Zhang, Ye Song, Li-Hai Tan

**Affiliations:** 1 School of Management, Jinan University, Guangzhou, China; 2 Department of Neurosurgery, Guangzhou Women and Children’s Medical Center, Guangzhou Medical University, Guangzhou, Guangdong, China; 3 Key Laboratory of CNS Regeneration (Ministry of Education), Guangdong-Hongkong-Macau Institute of CNS Regeneration, Jinan University, Shenzhen, China; 4 Center for Language and Brain, Shenzhen Institute of Neuroscience, Shenzhen, China; 5 Neuroscience and Neurorehabilitation Institute, University of Health and Rehabilitation Sciences, Qingdao, Shandong, China; 6 University International College, Macau University of Science and Technology, Macau, China

**Keywords:** animal research, expansion, language evolution, language theory, left inferior frontal gyrus, sub-region, vocal neuron, white matter connectivity

## Abstract

The evolutionary adaptation of the left inferior frontal gyrus is considered a crucial neural specialization supporting the emergence of human language. As a central node in the language network, it is linked to the temporoparietal cortex via both the ventral and dorsal pathways. These connections enable humans to combine a limited set of vocal elements into infinitely diverse, hierarchically structured sequences. Although homologous brain structures are also present in non-human primates, language remains a uniquely human faculty. This review synthesizes anatomical, functional, and connectivity evidence across species to trace the evolution of the left inferior frontal gyrus in support of language. We argue that language did not emerge from novel cortical areas, but through the gradual repurposing, expansion, and optimization of pre-existing fronto-temporal circuits. Human-specific innovations include vocal neuron specialization, volumetric expansion, strengthened connectivity of the arcuate fasciculus, and a functional shift within the left inferior frontal gyrus from motor control to syntactic processing. Finally, we discuss how lesion studies contribute to our understanding of the brain’s potential for language acquisition and its neurobiological constraints.

## 1 Introduction

Language is a unique human cognitive capacity among primates, particularly because human language can create infinite expressions through hierarchical and recursive syntax (Chomsky, 1957; Bohn et al., 2019; Matchin and Hickok, 2020; Altarriba and Basnight-Brown, 2022). While many species exhibit complex vocal behaviors, only human language integrates semantic, phonological, and syntactic components into a generative, open-ended linguistic system (Everaert et al., 2015; Fitch, 2020; Fujita and Fujita, 2022). For example, “the cat [that chased the mouse [that stole the cheese [that came from the farm [that …]]]] is running.” This integration depends heavily on the left inferior frontal gyrus (LIFG) (Hagoort, 2005; Pallier et al., 2011; Zhu et al., 2022).

Anatomically, the LIFG is comprised of three subregions: the pars opercularis, pars triangularis, and pars orbitalis (Udden and Bahlmann, 2012). In the human neuroimaging literature, they are often used to reflect the locations of Brodmann areas 44, 45, and 47, respectively. Although the correspondence between sulci used to define the morphological regions and their underlying cytoarchitectonic borders is imprecise (Zilles and Amunts, 2010; Tremblay and Dick, 2016; Sprung-Much and Petrides, 2018), we retain the BA terminology here to ensure a consistent framework for cross-species comparison, as many comparative neuroanatomical studies rely on these cytoarchitectonic definitions to establish homology between humans and NHPs (Petrides and Pandya, 2009; Frey et al., 2014; Palomero-Gallagher and Zilles, 2019; Hopkins et al., 2022). Among these, areas 44 and 45 constitute the classical Broca’s area, a pivotal region for voluntary speech production and orofacial motor sequencing (Rauschecker, 2018; Trettenbrein et al., 2021). Beyond language, these subregions also support general multimodal processes such as arbitrary visuomotor mapping, motor sequencing, and imitation (Levy, 2024). Functionally, area 44 is primarily engaged in syntactic encoding and regulating articulatory gestures through its connections with the ventral premotor cortex (Vigneau et al., 2006; Aglieri et al., 2018; Chen et al., 2021). In contrast, areas 45 and 47 support selective retrieval and controlled access of semantic information (Lyu et al., 2019; Sprung-Much and Petrides, 2020; Hodgson et al., 2021; Jasinska et al., 2021; Cordeau et al., 2023). Structurally, the LIFG connects to temporoparietal regions via dorsal (e.g., the arcuate fasciculus) and ventral (e.g., the uncinate fasciculus) pathways, forming the core of the language network (Hagoort, 2005; Friederici, 2011; Fedorenko et al., 2024).

In non-human primates (NHPs), comparative studies have identified homologues of Broca’s area and analogous frontal cortical networks involved in vocal control (Petrides and Pandya, 2002; Petrides et al., 2012; Hopkins, 2022; Amiez et al., 2023). For example, human area 44 corresponds to macaque area 44, which is located in the fundus of the inferior arcuate sulcus. Human area 45 corresponds to macaque area 45 on the anterior bank of the arcuate sulcus, while human area 47 corresponds to macaque area 47/12 on the ventrolateral surface (Petrides et al., 2012; Frey et al., 2014; Gavrilov and Nieder, 2021). However, despite these shared substrates, NHPs have not developed a human-like language system. One possible explanation lies in the neurofunctional specialization of Broca’s area and its associated white matter tracts in humans (Levy, 2024).

While the role of the LIFG in language evolution is a long-standing topic, recent advancements in high-resolution neuroimaging and comparative connectomics allow for a more granular re-examination of this question. For instance, Wang et al. (2020) provided direct evidence comparing the structural and functional connectivity patterns of the LIFG in humans and macaques. Results revealed significantly higher functional balance, increased low-frequency fluctuation amplitude, strengthened interregional coupling, and greater myelination of white matter tracts in the human LIFG, all of which likely contribute to the efficiency and complexity of human language processing. Additionally, current literature lacks a comprehensive framework that integrates these macro-scale imaging findings with clinical and cellular evidence. Synthesizing these multi-level datasets is essential to move beyond descriptive theories toward a mechanistic understanding of LIFG specialization.

**FIGURE 1:**
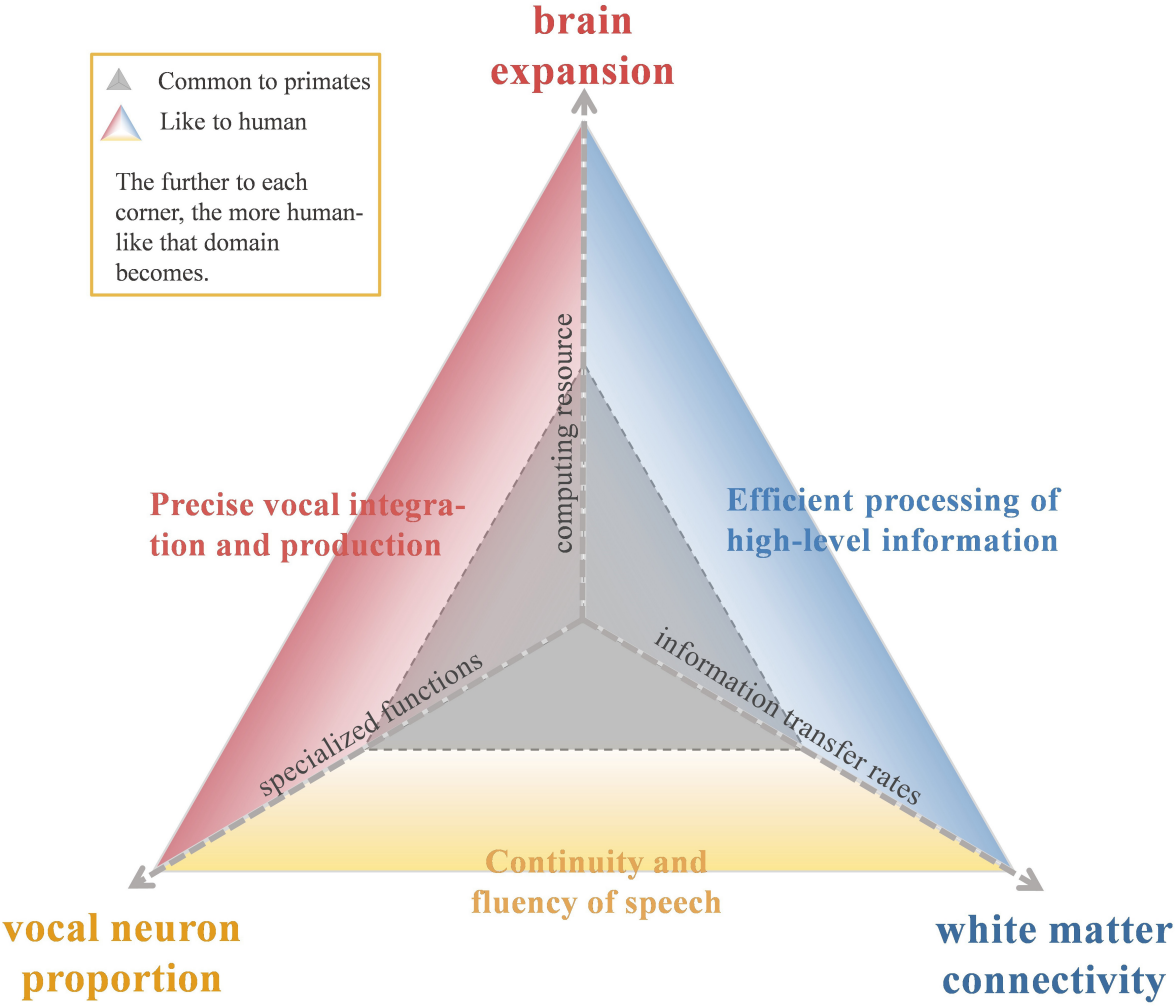
A multidimensional framework for tracing the evolution of the left inferior frontal gyrus (LIFG). The diagram illustrates the evolutionary comparison of the LIFG across three dimensions: (1) brain expansion (top axis), (2) vocal neuron proportion (bottom-left axis), and (3) white matter connectivity (bottom-right axis). The central gray triangle represents the shared neurobiological baseline common to non-human primates. The outward expansion (colored zones) depicts the human-specific evolutionary trajectory. As the axes extended outward from the center, they represent the enhancement of these biological substrates. These structural adaptions facilitate the emergence of unique human linguistic capabilities, specifically supporting precise vocal integration, efficient high-level processing, and speech fluency.

In this review, we systematically compare findings of the LIFG in humans and NHPs across three dimensions: neuronal activity, brain expansion, and white matter connectivity. As illustrated in Figure 1, this comparison visualizes how human-specific adaptations in these domains (outer triangle) have expanded from a shared primate baseline (inner triangle) to support the computational efficiency, articulatory precision, and fluency required for language. By integrating findings from neuroimaging, anatomy, and electrophysiology, we propose that language emergence did not rely on entirely novel brain regions or functions, but rather on the gradual adjustment of ancestral systems in response to social and communicative demands (Fisher and Marcus, 2006; Huybregts, 2019; Xu et al., 2020; Hage, 2024). We also discuss neural plasticity, examining how the divergent effects of LIFG lesions in humans and NHPs reveal the region’s evolutionary functional specialization. This perspective not only deepens our understanding of language origins but also offers translational insights for the diagnosis of aphasia subtypes, the prognostication of recovery outcomes, and the planning of targeted rehabilitation strategies. Table 1 summarizes representative empirical questions and approaches related to the LIFG and language evolution covered in this review.

**TABLE 1:** Empirical approaches to the role of LIFG in language evolution.

Research dimension	Empirical question	Methodology	References
Neurons and vocal control	Voluntary control of vocal signal as evidence for intentional communication	Behavioral studies of primate calls for predators, food, or social relationships; analysis of call rhythmicity; electrophysiological recordings of vocalization neurons	Hage and Nieder, 2013; Gavrilov et al., 2017; Loh et al., 2017; Crockford, 2019; Pomberger et al., 2019
	Call combinations	Experimental studies of context-dependent sequential call combinations	Girard-Buttoz et al., 2022; Bortolato et al., 2023b; Leroux et al., 2023
Anatomy and cortical expansion	Volume expansion and subregional differentiation of BA44	Cross-species cytoarchitectonic comparisons; MRI structural analyses	Schenker et al., 2010; Gallardo et al., 2023
Connectivity and network reorganization	Differential use of dorsal and ventral pathways	DTI tractography and tract tracing studies	Eichert et al., 2019; Barrett et al., 2020; Xia et al., 2021; Becker et al., 2022; Wu et al., 2022
Functional specialization and neural reuse	Functional migration from action to language	fMRI task studies; histological and cortical registration analyses of BA44/45; anterograde and retrograde tracing in macaques	Binkofski et al., 2000; Korponay et al., 2020; Papitto et al., 2020; Heckner et al., 2021; Gallardo et al., 2023
	Shift from sequence processing to hierarchical syntax	Artificial grammar learning experiments in NHPs	Fitch and Hauser, 2004; Ferrigno et al., 2020; Malassis et al., 2020
	Social interaction experience increases call sequence complexity	Field recordings and acoustic analyses with statistical modeling	Bründl et al., 2021; Bortolato et al., 2023a
Brain plasticity	Functional reorganization after LIFG lesions	Longitudinal studies of stroke recovery and right-hemisphere homolog compensation	Saur et al., 2006; Gajardo-Vidal et al., 2021

## 2 Retrieval strategy

We conducted a narrative literature review using PubMed and Web of Science. Keyword combinations covered four major domains: anatomy (“LIFG,” “Broca’s area,” “ventrolateral prefrontal cortex”), evolution and species (“language evolution,” “non-human primates,” “macaque,” “marmoset,” “chimpanzee”), neurobiology (“vocal neurons,” “cortical expansion,” “connectivity,” “white matter tracts”), and neural plasticity (“neural reuse,” “aphasia”). Inclusion criteria required that articles (a) be primary peer-reviewed research published in the English language, and (b) provide physiological, anatomical, or comparative imaging evidence relevant to the functional organization or evolution of the LIFG. We drew primarily on studies between 2020 and 2025, but included earlier, seminal studies as well. This time window was selected to capture the latest advancements in high-resolution neuroimaging, which have significantly refined our understanding of interspecies differences. Preprints offering novel empirical data were considered selectively.

## 3 Volitional vocal control in non-human primates

Volitional control of sound output is an indispensable prerequisite in language evolution (Hage and Nieder, 2016; Loh et al., 2017; Cheney and Seyfarth, 2018; Rauschecker, 2018; Nieder and Mooney, 2020; Jafari et al., 2023). Empirical evidence has shown that both New World monkeys (e.g., marmosets) and Old World species (e.g., chimpanzees) can produce context-driven vocalizations and flexibly switch between call types (Crockford, 2019; Pomberger et al., 2019; Girard-Buttoz et al., 2022). Furthermore, comparative analyses suggest that the articulatory capacities of great apes may be closer to human speech than previously thought. For example, great apes can produce sounds similar to voiced vowels and voiceless consonants (Boë et al., 2019; Grawunder et al., 2022; Lameira and Hardus, 2023; Lameira et al., 2025). Recent analyses of vocalizations in captive chimpanzees indicate that they can even produce syllabic utterances resembling words such as “mama” (Ekström et al., 2024). Moreover, behavioral studies indicate that chimpanzees can organize vocal units into structured sequences (Girard-Buttoz et al., 2022; Bortolato et al., 2023a; Leroux et al., 2023). These findings argue that great apes possess the requisite vocal control for language production, and their combinatorial capacities – while not fully generative – lay the foundation for an evolutionary continuum toward open-ended language (Girard-Buttoz et al., 2025).

In terms of neuronal activities, vocal production in NHPs primarily engages the ventrolateral prefrontal cortex (vlPFC) and premotor areas (Gifford et al., 2005; Romanski et al., 2005; Miller et al., 2015; Loh et al., 2017; Zhao and Wang, 2023). Specialized “volitional vocalization neurons” in these regions exhibit selective pre-vocal activity (Gavrilov and Nieder, 2021; Grijseels et al., 2023). In macaques, about 12% (54/454) of randomly sampled vlPFC neurons respond selectively to conspecific calls (Hage and Nieder, 2015). Compared with the anterior cingulate cortex (ACC) and pre-supplementary motor area (preSMA), the vlPFC has the highest proportion of vocally active neurons (33.4%, 180/545) and shows the strongest correlation with vocal amplitude and latency (Gavrilov et al., 2017). Functional distinctions also emerge between subregions. In rhesus monkeys, neurons in areas 44 and 45 are more active before vocalizations and may encode communicative intent, whereas neurons in the premotor cortex (area 6) primarily support motor execution of vocal output (Hage and Nieder, 2013; Loh et al., 2017; Hage, 2018).

Despite similarities, neural specialization in NHPs appears qualitatively and quantitatively distinct from that in humans. First, the coding precision is limited. For example, Plakke et al. (2013) reported that while 20% of vlPFC neurons (areas 12/47 and 45) were selective for vocalization type, their classification accuracy was modest (42%). Second, the distribution of these neurons differs: Hage and Nieder (2013) found that 27% of vocalization-related neurons were located in area 45, compared with 9% and 8% in areas 44 and 6, respectively. Similarly, Gavrilov and Nieder (2021) found that 20% of vocalization-evoked neurons were in the premotor area (area 6), 24% in area 45, and 24% in area 44. Additionally, most NHP vocalization-related neurons support basic acoustic modulation, with limited evidence for higher-order linguistic processes such as syntax (Romanski et al., 2005).

In contrast, the human vlPFC is anatomically more complex and crucial for translating communicative intent into hierarchically structured sequences (Hillert, 2021). It serves as a hub for multi-level processing, forming a robust network with the insula, motor cortex, and sensorimotor cortex (Long et al., 2016; Basilakos et al., 2018; Hickok et al., 2023; Willett et al., 2023). The “internal command apparatus” hypothesis (Archakov et al., 2020) proposes that human language generation relies on an integrated module involving Broca’s area, specifically area 44, to coordinate intended vocal output with the speech motor network (Long et al., 2016). Clinically, understanding how these specific neuronal populations encode communicative intent provides a neurophysiological baseline for modeling the mechanisms of voluntary speech initiation. Recent intracortical recording studies in paralyzed patients have further illuminated this functional architecture: Willett et al. (2023) found that while the ventral premotor cortex (Area 6v) contains rich articulatory representations sufficient to drive speech brain–computer interfaces (BCIs), area 44 appears less involved in immediate motor execution. The downstream motor regions retain the “executable” code for vocalizations, likely freeing the LIFG to specialize in high-order planning. Future research should employ high-resolution neural techniques (e.g., single-neuron recording, optogenetics, and neural tracking techniques) to systematically compare encoding mechanisms across species at the cellular level (Castellucci et al., 2022).

## 4 Language and brain size

Language acquisition imposes high metabolic demands and is historically correlated with brain size (Schoenemann, 2006). As brain size expands, the absolute number of neurons increases proportionally (Palomero-Gallagher and Zilles, 2019), boosting computational power and supporting fine-grained cognitive functions (Graic et al., 2020). Recent comparative analyses suggest that human cortical growth was driven by selective pressures rather than passive scaling. Smaers et al. (2021) reported that great apes (e.g., chimpanzees) exhibit a low slope of brain-body expansion (b ≈ 0.23) and that hominins were the only group showing a significantly positive slope (*b* = 1.10). These selective expansion rates are closely linked to the emergence of more complex socio-cognitive and linguistic functions (Schwartz et al., 2023; Vanderhaeghen and Polleux, 2023).

Anatomically, this expansion appears non-uniform, showing a predilection for high-order association cortices. Donahue et al. (2018) provided precise measurements, reporting that human prefrontal cortex (PFC) gray matter volume is 1.9 times that of macaques and 1.2 times that of chimpanzees. PFC white matter is 2.4 and 1.7 times larger, respectively. Compared to more evolutionarily conserved areas like the primary visual cortex (V1), the PFC exhibits pronounced anisotropic scaling, suggesting it grows at a disproportionately high rate. Within the IFG, subregions also exhibit distinct expansion patterns. Schenker et al. (2010) reported that the left area 44 in humans is 6.6 times larger than in chimpanzees, and area 45 is 6.0 times larger. These expansions make Broca’s area one of the most expanded cortical regions in the human brain. Gallardo et al. (2023) further validated this finding by comparing cytoarchitectural atlases of area 44 and area 45 between species. Their analysis showed that the left area 44 is the most disproportionately enlarged subregion in humans, expanding more anteriorly. When mapped onto a standardized cortical surface, human area 44 was 1.42 times larger than chimpanzees in the left hemisphere and 1.16 times larger in the right. In contrast, area 45 showed smaller differences (1.02 times in the left hemisphere, 1.35 times in the right). This specific enlargement likely reflects the brain responds to ecological and social environmental demands by altering the relative sizes of different brain regions (Dunbar and Shultz, 2007; Van Schaik et al., 2012; DeCasien and Higham, 2019).

However, the “Frontal Expansion Hypothesis” is not without controversy, and findings often depend on the methodological approach employed. For instance, Gabi et al. (2016), using isotropic fractionation to quantify neuronal and non-neuronal cells, reported only modest differences between humans and macaques. These differences were observed in PFC gray matter (10% vs. 7.6%), white matter (5.5% vs. 4.5%), and total cortical neuron counts (8% vs. 7.35%). These findings suggest that PFC expansion may not be as pronounced as commonly assumed when scaled against the rest of the cortex. Moreover, exaggerating the general disproportionate growth of the human prefrontal cortex may lead to false inferences. An overall larger prefrontal brain volume is not unique to humans; some non-human prosimian and anthropoid primates also have a higher ratio of frontal gray matter to total cortical volume than humans (Barton and Venditti, 2013; Barton and Montgomery, 2019).

Crucially, there is no simple linear causality between brain size and language. For instance, children with primary microcephaly can also achieve many developmental milestones despite reduced brain volume (Woods et al., 2005; Rilling, 2014). This suggests that while a critical mass of neural substrate is necessary, gross volumetric expansion alone is insufficient to account for the emergence of complex language functions, such as syntax.

In sum, while the LIFG has undergone significant volumetric expansion, this expansion provides only the “hardware” capacity. The emergence of language likely required the functional optimization of this expanded tissue through enhanced connectivity, rather than sheer size alone.

## 5 Brain connections involving LIFG optimized with neural networks

The brain does not operate in isolation; instead, it supports coordinated functional interactions across regions through white matter connections (Friederici et al., 2017; Sanchez et al., 2023). Language acquisition depends not only on the expansion of cortical “raw material” (e.g., brain volume), but also on targeted modifications in specific neural pathways. A widely accepted view suggests that language is a recent evolutionary event, and has involved shared and fine-tuned neural circuits between humans and NHPs (Wilson et al., 2017; Fitch, 2018). Within these circuits, the LIFG-centered language network in humans shows marked structural specialization, with optimized connectivity across both dorsal and ventral pathways (Angel Rivas-Fernandez et al., 2021; Becker et al., 2025). We summarize the functional roles and evolutionary status of these major tracts in Table 2.

**TABLE 2:** Key language-related white matter tracts of the LIFG: function, clinical significance, and evolutionary status.

Tract	Primary function in language	Clinical deficit	Evolutionary status (Human vs. NHPs)
Arcuate fasciculus (AF)	Connects area 44 to posterior STG/MTG; supports auditory-motor mapping, syntactic integration, speech processing, and language production (Saur et al., 2008; Friederici, 2011).	Conduction aphasia; phonological errors and paraphasias; syntactical errors (Duffau et al., 2002; Fridriksson et al., 2010; Vidorreta et al., 2011)	Human-Specific Expansion. Robust in humans with deep projections into the MTG. In NHPs, it is anatomically restricted, terminating primarily in the STG (Balezeau et al., 2020; Becker et al., 2022; Hopkins, 2022; Eichner et al., 2024).
Superior longitudinal fasciculus III (SLF-III)	Connects the supramarginal gyrus to the premotor areas and LFG; supports syntactic, visuo-spatial processing and sensory-motor integration (Klingberg et al., 2000; Catani et al., 2005; Makris et al., 2005).	Speech arrest, dysarthria, and repetition errors (Galantucci et al., 2011; Maldonado et al., 2011; Chang et al., 2015)	Human-Specific Expansion. In humans, it shows substantially larger volume and an anterior extension into the inferior frontal gyrus (area 44/45). Chimpanzee SLF III, by comparison, terminates primarily in the ventral premotor cortex (Hecht et al., 2015).
Inferior fronto-occipital fasciculus (IFOF)	Connects the inferior frontal cortex and dorsolateral prefrontal cortex to the posterior temporal and occipital lobes; supports visual and semantic processing (Turken and Dronkers, 2011; Dick and Tremblay, 2012)	Semantic paraphasias (Duffau et al., 2005; Leclercq et al., 2010; De Witt Hamer et al., 2011)	In humans, it expands posteriorly into the occipital lobe, a projection absent in macaques, whose comparable functions may immediate by the EmC (Thiebaut de Schotten et al., 2012; Wang et al., 2016).
Uncinate fasciculus (UF)	Connects the frontal operculum and the anterior STG/STS; supports semantic processing and sound recognition (Diehl et al., 2008; McDonald et al., 2008)	Naming deficits (anomia) and semantic impairments (Papagno et al., 2011)	Conserved. Anatomy is highly conserved between humans and NHPs (Dick and Tremblay, 2012; Thiebaut de Schotten et al., 2012).
Extreme capsule (EmC)	Connects posterior temporal and occipital areas to vlPFC via the UF and IFOF; supports semantic processing and phonological working memory (Makris and Pandya, 2009; Saur et al., 2010).	Semantic paraphasias; transcortical sensory aphasia (Boatman et al., 2000; Duffau et al., 2005; Saur et al., 2008)	The primary ventral language pathway in macaques. In humans, functionally integrated with or overshadowed by the expanded IFOF (Makris and Pandya, 2009; Thiebaut de Schotten et al., 2012).

The traditional dorsal pathway, which targets area 44, connects the posterior superior temporal gyrus and the inferior parietal cortex through the superior longitudinal fasciculus (SLF) or arcuate fasciculus (AF), supporting phonological structures mapping and core syntactic computation (Saur et al., 2008; Friederici, 2011; Rauschecker, 2018; Sanchez et al., 2023). Anatomically, the most striking evolutionary innovation lies in the dorsal pathway, particularly the AF. In humans, the AF is robust and densely packed (Barrett et al., 2020). It links area 44, superior temporal gyrus (STG) (Glasser and Rilling, 2008; Dick and Tremblay, 2012), and extends deeply into more posterior regions of the MTG and inferior temporal areas–an anatomical pattern previously considered unique to humans (Rilling et al., 2008).

In contrast, comparative studies show that in macaques, the AF mainly connects the frontal cortex with the superior temporal cortex and lacks fibers that extend to the middle or inferior temporal lobes (Eichert et al., 2019). While recent high-resolution diffusion imaging in chimpanzees suggests the presence of a nascent dorsal projection toward the MTG (Balezeau et al., 2020), its connectivity strength is quantitatively negligible compared to humans (Sierpowska et al., 2022). Becker et al. (2025) quantified this shift: AF-MTG connectivity is 6.3 times that of AF-STG in humans, versus 1:14.3 in chimpanzees. This expansion cannot be explained solely by cortical enlargement or spatial reallocation; rather, it suggests a qualitative reorganization to support the rapid transmission of phonological and syntactic information.

Furthermore, the human AF shows pronounced left lateralization, which is consistent with the well-known left-hemispheric dominance in language processing (Balezeau et al., 2020; Hopkins, 2022; Eichner et al., 2024). In contrast, chimpanzees and macaques do not show consistent population-level lateralization of the AF (Xia et al., 2021; Hecht et al., 2025).

In parallel, humans possess a robust and functionally important ventral stream, including the extreme capsule (EmC), uncinate fasciculus (UF), and inferior fronto-occipital fasciculus (IFOF). Unlike the dorsal stream, which underwent massive reorganization, the ventral stream represents an evolutionarily conserved foundation. It connects the anterior LIFG (area 45/47) to the anterior superior temporal cortex, supporting semantic mapping and high-level conceptual integration (Anwander et al., 2007; Bornkessel-Schlesewsky et al., 2015; Wu et al., 2022; Rolls et al., 2023). The key evolutionary distinction is that humans leverage this system as an essential component of a complex, dual-stream language faculty, whereas NHPs rely almost exclusively on this ventral system for communication (Berwick et al., 2013; Eichert et al., 2019; Balezeau et al., 2020; Becker et al., 2022; Friederici, 2023).

Evolutionary pressures have also driven an increased connectivity density. Smaers et al. (2010) analyzed 18 primate species and found that prefrontal white matter exhibits the steepest scaling relative to brain size (*b* = 1.33), with stronger correlations to total brain volume than non-prefrontal white matter (R^2^ = 0.338 vs. 0.069). Previously, Schoenemann et al. (2005) had reported that prefrontal white matter constitutes 10.9% of total brain white matter in humans, compared to 7.7% in great apes and 7.2% in monkeys. Across all human samples, prefrontal white matter volume exceeded values predicted from non-human primate scaling trends by an average of 41% (16.5 ml). This “white matter dominance” supports the hypothesis that human cognition relies on the enhanced efficiency of long-range networks.

Nevertheless, current imaging techniques face methodological hurdles. Specifically, while diffusion tensor imaging (DTI) and high-resolution MRI enable whole-brain structural mapping, their resolution and signal-to-noise ratio are insufficient for tracking fine-grained fiber trajectories (Jones et al., 2013; Thomas et al., 2014; Reveley et al., 2015). Furthermore, while invasive methods such as autoradiography provide higher anatomical accuracy, these methods are also constrained by section thickness and sampling density (Albanese and Chung, 2016; Xu et al., 2021). To address these challenges, future studies should integrate multi-scale imaging approaches (e.g., quantitative MRI, *in vivo* tissue imaging) with large-scale connectome datasets to more precisely characterize interspecies differences in the structure of language-related pathways (Weiskopf et al., 2021; Chauvel et al., 2023).

## 6 Hypotheses of language evolution

**FIGURE 2:**
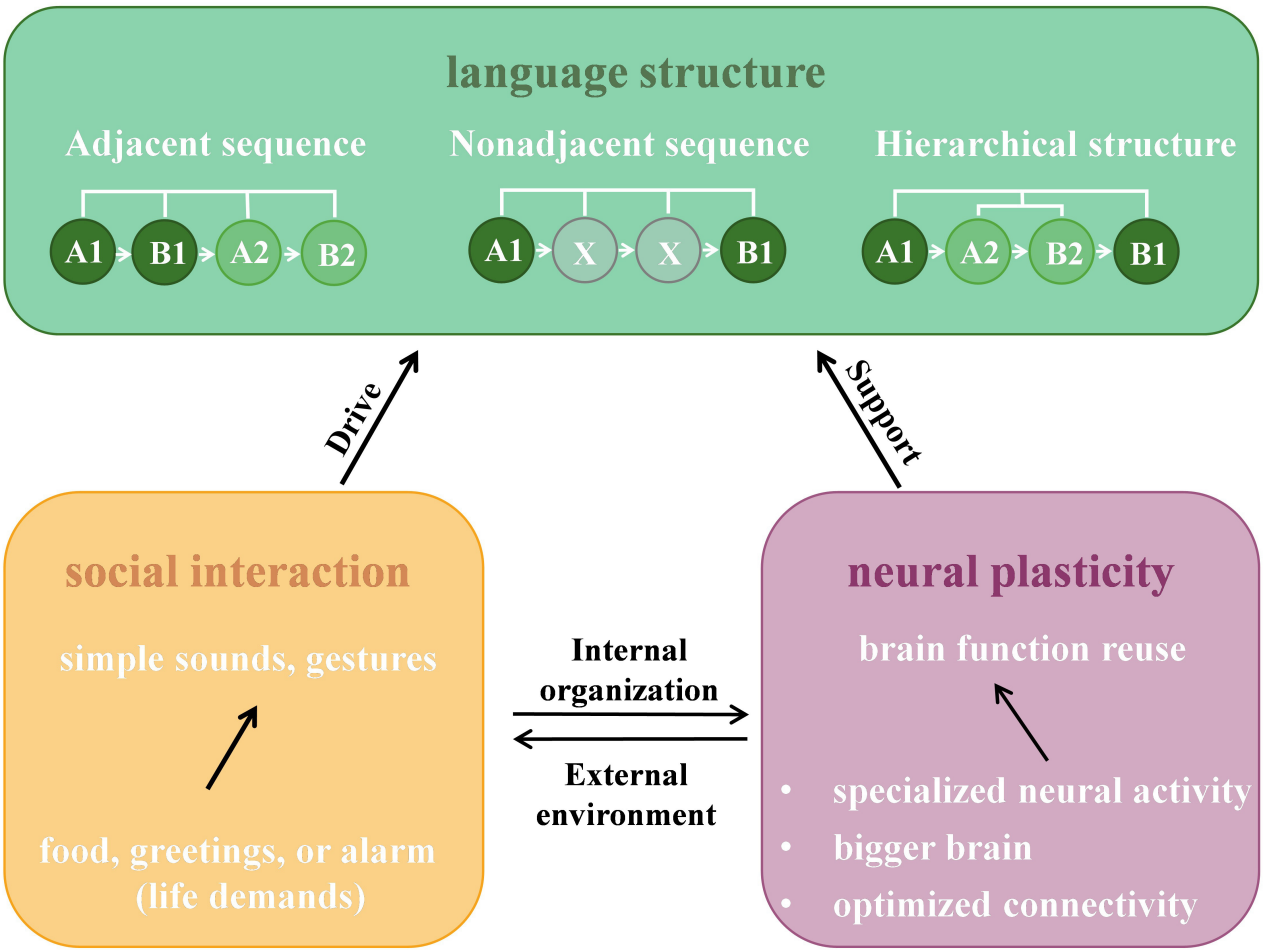
An integrated bio-social framework for the emergence of language structures. The schematic illustrates how the interaction between external environmental pressures and internal neurobiological reorganization drives the evolution of language complexity. As social complexity increases, simple sounds and gestures become insufficient, creating selective pressure for more sophisticated communication systems (yellow panel). To meet these demands, the brain repurposes existing circuits and areas for novel functions (purple panel). The green panel depicts the resulting structural hierarchy: while NHPs can process Adjacent Sequences (linear A1→B1) and limited Non-adjacent Sequences (A1→*X*→B1), the convergence of social drive and neural support enables humans to master Hierarchical Structures (recursive nesting, e.g., [A1→(A2→B2)→B1]). The example “The cat [that ate the mouse] is sleeping” illustrates this recursive capability, which remains a uniquely human faculty supported by the specialized LIFG network.

Although the LIFG of NHPs shares similar connectivity patterns and some functional features with humans, it remains puzzling why they have not evolved language-like systems (Levy, 2024). Hage (2024) suggests that the brains and vocal tracts of NHPs are anatomically “speech-ready,” showing features like vocal rhythmicity and task-driven vocal modulation (Fitch et al., 2016; Risueno-Segovia and Hage, 2020; Lameira and Hardus, 2023). NHPs can even learn simple linear sequences and basic combinatorial rules, such as adjacent relationships (Watson et al., 2020). However, studies have confirmed that NHPs remain limited in processing recursive hierarchical structures and long-distance dependencies (Hauser et al., 2002; Fitch and Hauser, 2004; Malassis et al., 2020; Girard-Buttoz et al., 2022). The emergence of language may require the convergence of external pressures and internal neurobiological adaptations. As illustrated in Figure 2, this process is driven by the interplay between the external environment (social interaction) and internal organization (neural plasticity).

The “social complexity hypothesis” posits that increasing communicative demands in social groups create selective pressure (“Drive”) for a more complex signaling system (Seyfarth and Cheney, 2014; Cheney and Seyfarth, 2018). Simple calls and gestures eventually become insufficient, necessitating the evolution of structural complexity (Pougnault et al., 2022; Bortolato et al., 2023b; Grampp et al., 2023). To meet these external demands, the brain must provide the necessary support through neural plasticity (Figure 2, purple panel). The neural reuse theory proposes that language did not arise from entirely new brain regions, but rather through the reuse and functional reorganization of existing neural circuits (Zilles and Amunts, 2018). Brain regions are often multifunctional: pre-existing neural circuits can be reorganized for novel cognitive tasks without losing their original functions (Anderson, 2007, 2010; Dehaene and Cohen, 2007). As synthesized in this review, this reorganization manifests across three dimensions: specialized neural activity, volumetric expansion, and optimized white matter connectivity.

Broca’s area within the LIFG may have originally participated in motor planning and tool use. As social pressures increased, these regions – coding for hierarchical motor goals – were exapted to support the hierarchical structures of language (Nishitani and Hari, 2000; Kemmerer, 2022). Extensive neuroanatomical evidence supports this Motor-to-Syntax transition. Human area 44 exhibits a clear dorsoventral subdivision: the dorsal part (area 44d), adjacent to area 6, is mainly involved in motor processing, while the anterior-ventral part (area 44v) is specialized for syntactic operations (Amunts et al., 2010; Hamzei et al., 2016; Loh et al., 2020; Papitto et al., 2020; Heckner et al., 2021; Zaccarella et al., 2021; Neef et al., 2023). Invasive tracing studies in macaques also reveal a comparable topological organization. For example, Korponay et al. (2020) found that the rostral portion of area 44 predominantly projects to the caudate nucleus and receives input from non-motor prefrontal areas, whereas the caudal portion projects to the putamen and integrates with motor-related input from area 6VR. Gallardo et al. (2023) recently quantified the overlap of action- and syntax-related regions within area 44 in both chimpanzees and humans, showing that the chimpanzee area 44 overlaps more with action-related posterior regions and less with anterior syntax-related areas. The fundamental syntactic operations may have evolved from motor sequencing programs, with language inheriting the neural architecture of action chains (Pulvermueller, 2018; Thibault et al., 2021; Zaccarella et al., 2021; Fujita and Fujita, 2022; Gallardo et al., 2023).

In summary, human language evolution was likely driven by the mosaic evolution of the LIFG. The changes in the natural environment necessitate more complex behavioral, social, and communicative capacity that shapes the complexity of neural development in all species, as well as humans. The synergy between social demands and neural reorganization allowed humans to transcend simple adjacent sequences to master the hierarchical dependencies required for open-ended communication. Future studies should integrate cross-species neuroanatomical comparisons with behavioral and ecological data to clarify the neural origins of language.

## 7 Neural plasticity and language recovery

Neuroplasticity after language-related brain damage provides a crucial foundation for understanding the dynamic characteristics of brain function (Hartwigsen and Saur, 2019). These neurobiological mechanisms enhance our understanding of how the brain acquires and restores language, and also highlight constraints that may have influenced language evolution across species (Stengel et al., 2022).

In humans, stroke-induced Broca’s aphasia is often the result of ischemia in the LIFG, causing impairments in speech fluency, rate, and grammatical processing (Grossman and Irwin, 2018; Lau et al., 2021). Saur et al. (2006) described three stages of language recovery: (1) initial reduction of activation in intact left-hemisphere language regions, (2) subsequent bilateral increase, especially in the right Broca-homologue, associated with functional improvement, and (3) eventual normalization of activation, reflecting system stabilization. Recovery may rely on the brain’s neurocomputational resilience through up-regulation of intact regions and recruitment of tissue around the lesion and right-hemisphere homologues (Stefaniak et al., 2020; Galaburda, 2022).

Recent lesion-symptom mapping studies challenge the classical view of Broca’s area as the central hub for long-term speech production. Gajardo-Vidal et al. (2021), studying 134 stroke survivors with focal left frontal lesions, found that damage to the anterior arcuate fasciculus (aAF) predicted persistent speech deficits more strongly than damage to area 44 or 45. More than 70% of cortical damage on verbal output was mediated by concurrent white matter damage, especially to the aAF. When aAF integrity was considered, damage to area 44 no longer predicted speech performance. These findings have direct clinical implications for stratifying prognosis and treatment. The integrity of the AF may emerge as a decisive biomarker of recovery potential. Patients with preserved AF connectivity are prime candidates for restorative therapies aimed at reactivating perilesional circuits. In contrast, transection of the AF predicts a high likelihood of persistent deficits. For these patients, therapy should shift early toward compensatory strategies, such as recruitment of right-hemisphere homologues or reliance on the intact ventral pathway (Yeatman and Feldman, 2013).

Evolutionarily, this distinct vulnerability highlights the “cost” of specialization. Although vocal production in non-human primates robustly engages the vlPFC, with a high proportion of vocally active neurons, lesion evidence suggests that this region is not strictly necessary for spontaneous call production. In contrast to humans, damage to the vlPFC in monkeys has little impact on vocal behavior (Jürgens, 2002; Hillert, 2021), indicating functional divergence of homologous regions across primate species.

Despite these insights, clear mechanistic explanations of reorganization remain to be established. Current studies mainly examine activation changes, lacking detailed insights into micro-structural regeneration–specifically, how synapse trajectories reorganize, how receptor densities adapt, and how dendritic spines remodel after LIFG injury. Future research should integrate high-resolution neuroimaging, cellular tracing, and molecular neuroscience techniques to capture the full landscape of neural regeneration, circuit reorganization, and synaptic network adaptation; such an integrated study may offer multi-scale insights into language evolution and rehabilitation.

## 8 Conclusion

Human language acquisition is mediated by the LIFG, which functions as a critical hub for language through its connections with dorsal and ventral pathways, supporting the coordination of phonological, syntactic, and articulatory processes. From an evolutionary perspective, this review synthesizes evidence from neuronal activity, brain expansion, and cross-species connectivity to highlight the unique role of the human LIFG in the emergence of language. While the precise evolutionary steps remain difficult to reconstruct, findings suggest that human language capacity arose through the gradual strengthening and specialization of these networks. This evolutionary perspective can also translate into clinical reality. By mapping the specific neural constraints that emerged during human evolution, we can gain a more precise biological framework for interpreting the mechanisms of language production and loss, thereby guiding the development of targeted rehabilitation strategies.
